# Systemic Inflammation in Sarcopenia Alter Functional Capacity in Thai Community-dwelling Older People: A Preliminary Observational Study

**DOI:** 10.2174/1874609815666220513141300

**Published:** 2022-08-04

**Authors:** Kornanong Yuenyongchaiwat, Chareeporn Akekawatchai

**Affiliations:** 1 Physiotherapy Department, Faculty of Allied Health Sciences, Thammasat University, 12120, Thailand;; 2 Research Unit for Physical Therapy in Respiratory and Cardiovascular Systems, Thammasat University, Pathumthani, Thailand;; 3 Medical Technology Department, Faculty of Allied Health Sciences, Thammasat University, Pathumtani, Thailand;; 4 Research Unit in Diagnostic Molecular Biology of Chronic Diseases Related to Cancer (DMB-CDC), Thammasat University, Pathumthani, Thailand

**Keywords:** Sarcopenia, inflammatory markers, functional capacity, older people, prevalence, community

## Abstract

**Background:**

Sarcopenia is linked to the loss of muscle mass in older adults, leading to impaired functional capacity and quality of life. In addition, this finding was recognized as an age-related chronic inflammatory process. We aimed to determine the relationship between sarcopenia, functional capacity, and inflammatory biomarkers and subsequent prediction of inflammatory biomarkers in older adults.

**Methods:**

A total of 126 women and men aged ≥ 60 years were enrolled. Participants were required to complete a handgrip dynamometer, 6-meter walk test, and bioimpedance analysis. Diagnosis was based on the definition of sarcopenia from the Asian Working Group for Sarcopenia 2019. Prior to performing a 6-minute walking test (*i.e.*, functional capacity testing), blood samples were drawn for a C-reactive protein (CRP) test.

**Results:**

A total of 12.70% were categorized as having sarcopenia. Significant differences in CRP and functional capacity between the sarcopenia and non-sarcopenia groups were found (*p* <.05). Older people with high CRP levels had significantly reduced functional capacity and slow gait speed.

**Conclusions:**

Poor functional capacity was associated with increased CRP levels, which might be due to the development of age-related inflammation. Older patients with sarcopenia may be at higher risk for functional decline.

## 1 INTRODUCTION

Sarcopenia is an age-related adverse health outcome in community-dwelling individuals. It has been pinpointed that sarcopenia is a process of a chronic inflammatory illness within the older population. In addition, older people with sarcopenia have been noted in several studies, whether in European or Asian countries [1-6]. The prevalence rate of sarcopenia among older people ranged from 5.5% to 25.7% using the Asian Working Group for Sarcopenia (AWGS) 2014 criteria [7] and from 1% to 33% using the European Working Group on Sarcopenia in Older People (EWGSOP) criteria [8], which was based on a variety of definition criteria for sarcopenia.

Important factors for diagnosis include guidelines (*e.g.*, EWSGOP, AWGS), instruments used to assess muscle mass (*e.g.,* dual-energy X-ray absorptiometry (DEXA) and bioelectrical impedance analysis (BIA)), and regional settings (*e.g.*, community, hospital) [9]. The prevalence of sarcopenia is projected to increase from 11.1% in 2016 to 12.9% in 2045 [9].

Older people with sarcopenia display low cardiovascular endurance (*i.e.*, poor functional capacity). Sarcopenic elderly had poor cardiovascular performance compared to the non-sarcopenic elderly. These results lead to poor quality of life and increased morbidity and mortality) [10]. Recent evidence indicates that chronic inflammation contributes to sarcopenia and that systemic inflammatory mediators affect muscle protein metabolism and muscle strength [11, 12]. Furthermore, C-reactive protein (CRP), an inflammatory mediator, has been reported in sarcopenic elderly, with some studies showing an association [13, 14], though one study revealed no association with sarcopenia [15]. Moreover, the mechanism of the inflammatory process in older people with sarcopenia remains unclear and controversial. Therefore, the present study aimed to determine the relationships and prediction of serum CRP levels in functional capacity and the components of sarcopenia among Thai older people.

## 2 MATERIALS AND METHODS

### 2.1 Participants

The study was conducted in accordance with the Declaration of Helsinki and the Ethics Human Committee of Thammasat University, according to the Declaration of Helsinki, the Belmont Report, CIOMS Guidelines, and the International Practice (ICH-GCP), approval number COA no. 023/2562. A cross-sectional study based on older people who lived in the community were invited to participate in community-based services. The study involved collecting blood samples, screening for sarcopenia, and performing functional capacity tests. Serum samples were tested for high-sensitivity C-reactive protein (hs-CRP) concentrations using an immunoturbidimetric method (CRP Vario test, ARCHITECT ci 8200 analyzer, Abbott Diagnostics, Abbott Park, IL, USA).

### 2.2 Evaluation

Based on the definition of the AWGS in 2019, muscle mass, muscle strength, and physical performance were assessed in older men and women aged ≥60 years. Bioimpedance analysis (BIA: Omron HBF-375 body composition monitor; Omron Healthcare Co., Ltd., Japan) was used to estimate skeletal muscle mass (SMM). The skeletal muscle mass index was obtained by dividing SMM by height (m^2^). Handgrip strength was assessed as muscle strength, which was measured by using a handheld dynamometer (T.K.K.5401 Grip ‐ D; Tokyo, Japan). Physical performance was measured using a 6-meter walk test, which assesses gait speed. In addition, the participants were classified based on the definition of AWGS criteria updated in 2019, which states these as the criteria for sarcopenia: low muscle mass (*i.e.*, less than 7.0 kg/m^2^ for males and less than 5.7 kg/m^2^ for females), plus slow gait speed (*i.e.*, less than 1.0 m/s for males and females) or and low handgrip strength (*i.e.*, less than 28 kg for males and less than 18 kg for females) [7]. Cardiovascular endurance was determined by functional capacity assessed using the 6-minute walk test, and then the walking distance was recorded [16].

### 2.3 Statistical Analysis

Statistical analyses were performed using IBM SPSS version 23.0. Normality distribution of the data was reported with the Kolmogorov–Smirnov goodness of fitness test. Descriptive data are presented as means, standard deviations and percentages. Chi-square test or *t*-test, where appropriate, was used to compare the sarcopenia and non-sarcopenia groups. Correlation analysis was performed to assess the association between high CRP levels and functional capacity. For this purpose, hs-CRP levels ≥ 3.0 mg/L were defined as high CRP levels [17, 18]. A *p*-value of <.05 was set as a statistical significance.

## 3 RESULTS

A total of 126 participants were enrolled between February and March 2021 during the COVID-19 pandemic in Thailand. Of these, 12.70% were diagnosed as having sarcopenia (7.94% female and 4.76% male participants). Older males showed a higher rate of diagnosis than older female participants (25.00% of 24 males and 9.80% of 102 females). However, no significant differences were observed between men and women (7.94% *vs*. 4.76%, *p*=.08). Sarcopenic elderly individuals had muscle mass decrease (4.78±0.77 kg/m^2^ *vs*. 6.07±1.18 kg/m^2^, *p* <.001), low physical performance (*i.e.*, gait speed: 1.03 ± 0.24 m/s *vs*. 1.24±0.19 m/s, *p*<.001), poor functional capacity (*i.e.*, 274.94±95.57 meters *vs.* 350.98±82.65 meters, *p*= .001), and high inflammatory biomarker levels (*i.e.*, hs-CRP: 4.75 ± 5.94 mg/L *vs*. 2.57 ± 3.70 mg/L, *p*= .046) compared with non-sarcopenic older individuals (Table **1**).

**Fig. (1):**
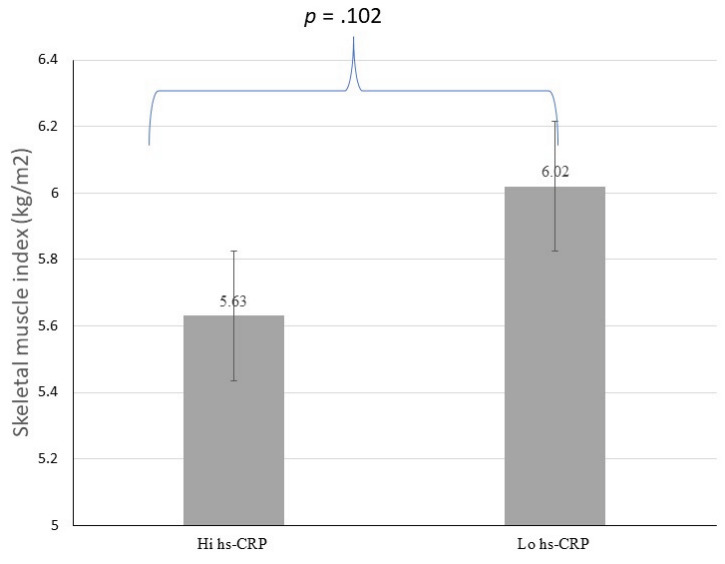
Difference of Skeletal muscle index according to hs-CRP.

**Fig. (2):**
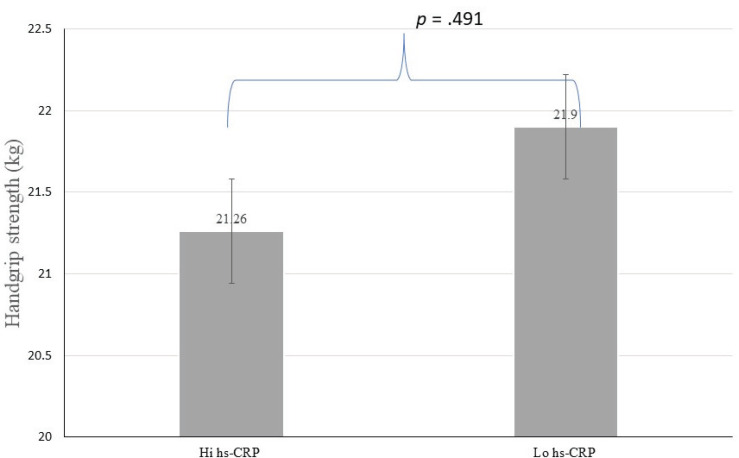
Difference of handgrip strength according to hs-CRP.

**Fig. (3):**
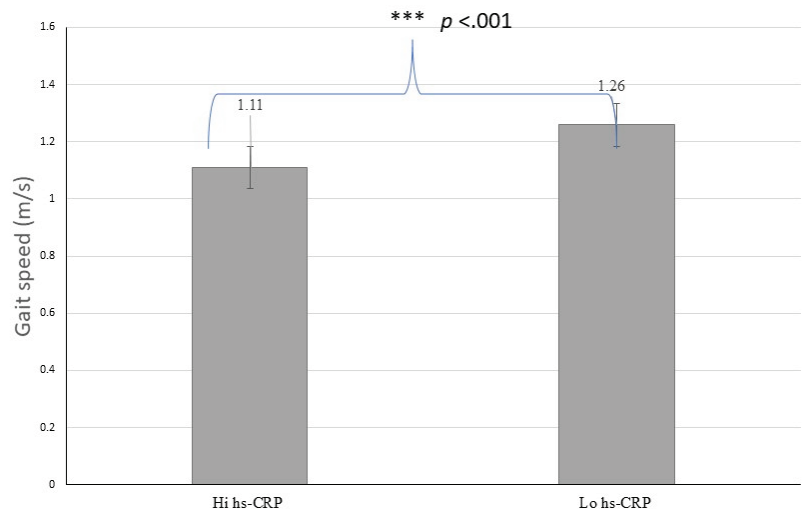
Difference of gait speed according to hs-CRP.

**Fig. (4):**
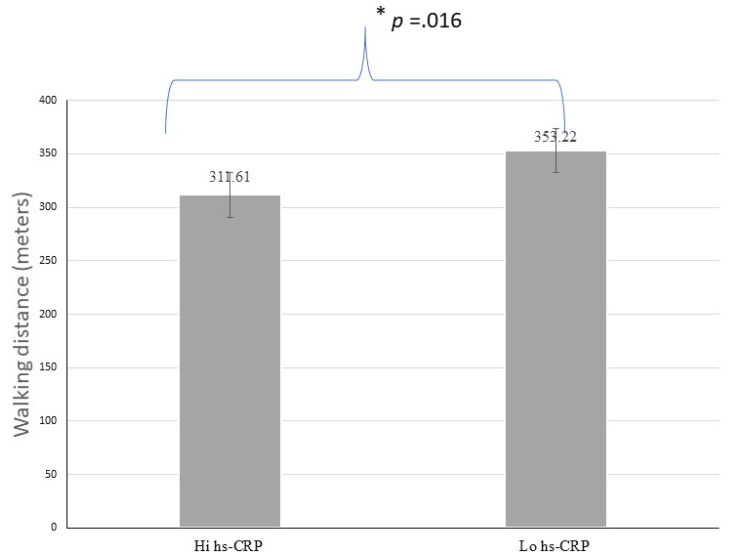
Difference of walking distance according to hs-CRP.

Analyzes were conducted using the hs-CRP levels with a cut-off < 3.0 mg/L, indicating a possible pathology of risk for cardiovascular events (Table **2**). There were no significant differences in age, sex, handgrip strength, and muscle mass index regarding the hs-CRP category (see Figs. **1** and **2**). Individuals with high hs-CRP levels had a slow gait speed and lower functional capacity than those with low hs-CRP levels (*p* <.05) (Figs. **3** and **4**).

Regarding sex differences, only female older adults who were categorized as having high hs-CRP had a low skeletal muscle index, slow gait speed, and poor functional capacity compared to those with low hs-CRP (Table **3**).

In addition, Spearman’s rank correlation revealed that high hs-CRP was negatively associated with gait speed (*r* = -0.310, *p* <.001). Further, it was inversely associated with walking distance (*r* = -0.268, *p* = .002). In other words, a high risk of cardiovascular events, defined as high hs-CRP, was associated with poor functional capacity (Table **4**).

## 4 DISCUSSION

The present study reported that individuals with sarcopenia had advanced age, decreased muscle mass (*i.e.*, skeletal muscle mass), low physical performance (*i.e.*, gait speed), and poor functional capacity (*i.e.*, 6-minute walk test) compared to those without sarcopenia. In addition, higher CRP levels were observed in the sarcopenia group than in the non-sarcopenia group. Reduced functional capacity and slow gait speed were associated with high hs-CRP levels (≥ 3.0 mg/L). These results revealed that only female older adults with high hs-CRP levels showed a lower gait speed, lower muscle mass, and poorer functional capacity, not males. A reduction in gait speed and poor functional capacity may be predictive of high hs-CRP; these results support that the inflammatory biomarker index (*i.e.*, high hs-CRP) could be used to identify the development of risk for sarcopenia.

The present study showed a significant association between the determination of sarcopenia status (*i.e.*, muscle mass index, gait speed, and handgrip strength) and CRP levels, which were associated with low gait speed, low muscle mass (only in female older adults), and high hs-CRP. Handgrip strength was not found to be associated. These findings were in line with previous studies that have found high CRP levels related to the component of sarcopenia (*i.e.*, slow gait speed, poor handgrip strength, low muscle mass) in the older people, such as lower muscle mass, decreased physical performance, and decreased muscle strength [18-20]. However, other studies have reported that high CRP levels were associated with a decrease in handgrip strength [14, 18]. Recently, a systematic review with meta-analysis with 14 cross-sectional studies indicated a relationship between hs-CRP levels and muscle strength (effect size = -0.22; 95%CI= -0.34 to -0.09) [21]. In contrast to those studies, 384 Chinese community-dwelling older people showed no association between sarcopenia and CRP levels [15]. Although the platelet-to-lymphocyte ratio (PLR) related to muscle mass and lymphocyte-to-monocyte ratio (LMR) was correlated with handgrip strength, no association with inflammatory biomarkers (*i.e.*, PLR, neutrophil-to-lymphocyte ratio (NLR), LMR, and CRP levels) has been reported [15]. Therefore, the mechanism of the inflammatory process in older people remains unclear and controversial. Some suggested the hypothesis that the mechanisms linking sarcopenia may be, in part, due to a decreased secretion of hormones (*e.g.*, endocrine changes, adrenal, thyroid hormones, catabolic hormones) that are involved in muscle atrophy, particularly type IIb [22-24]. Another possible mechanism is that neurodegenerative conditions occur within the motoneuron number or nuclear transport proteins [25, 26]. Thus, the mechanism linking sarcopenia might not be a specific pathophysiological pathway and may be explained by multisystemic changes in older people. Herein, we investigated the Thai community during the COVID-19 pandemic, which might lead to physical inactivity and social interaction because of social distancing; therefore, low physical activity might also be related to inflammatory markers [27].

Although the mechanistic link between CRP elevation and sarcopenia is still unclear, there are in vitro studies supporting the role of chronic elevation of CRP in aging-related diseases and muscle mass loss [15, 19]. Exposure of human myotubules to CRP resulted in a reduction in muscle protein synthesis rate, induction of activation pathways of cellular energy stress regulators, AMP-activated protein kinase (AMPK), and downregulation of factors controlling protein synthesis, Akt, and ribosomal protein S6 (rpS6) [19]. CRP might also be related to other illnesses, such as chronic disease or underlying inflammation; therefore, further studies are needed to determine other risk factors.

Interestingly, age-related decline in serum hs-CRP levels in the present study was not observed, with an average hs-CRP level of 2.85 ml/L at a mean age of 68.7 years. In contrast, previous studies found changes in age to be associated with inflammatory markers (*i.e.*, hs-CRP) [15, 28]. The serum levels of hs-CRP showed a tendency to increase with advancing age from 20 to 90 years, and a significantly higher difference in hs-CRP levels was observed in patients aged ≥ 65 years compared to those aged < 65 years [28]. In addition, in a meta-analysis including 32 cross-sectional studies, individuals with frailty (1,698 participants) and pre-frailty (8,568 participants) had significantly higher CRP levels compared to those without frailty (6,757 individuals) [29]. With a large older population (6,172 participants aged 60 years and above), high CRP levels were reported in participants with possible sarcopenia (defined as low muscle strength and/or decreased physical performance) compared to participants without sarcopenia [13]. These studies demonstrated that the elevations in CRP values were negatively associated with sarcopenia status and that CRP levels reflect inflammatory and aging biomarkers [30-32]. In addition, CRP plays an important role as a risk factor for age-related diseases such as cardiovascular disease, diabetes mellitus, hypertension, neurological conditions (*e.g.*, Parkinson’s disease, Alzheimer’s disease), and chronic kidney disease [15]. Older people in the present study had multiple chronic diseases, such as type-2 diabetes and hypertension. Furthermore, older adults with type-2 diabetes have shown high hs-CRP levels (data not shown). Therefore, comorbidity might be partly due to high CRP levels.

The association between functional capacity (*i.e.*, 6-minute walk test) and CRP, defined as decreased walking distance, was correlated with CRP in several patient populations, such as those with schizophrenia, heart disease, and chronic obstructive pulmonary disease (COPD), and in the healthy population [33-36]. CRP was inversely correlated with physical performance (measured using a 400-m walk test) in older people; higher physical fitness lowers CPR levels in older men adults (aged 74.6 years) [27]. In addition, low cardiorespiratory fitness was associated with high CRP levels in men [37]. In a cross-sectional study of older adults aged 70-79 years, high physical activity (measured using an interviewer-administered questionnaire from the previous 12 months) was associated with low levels of CRP; lack of exercise was related with high CRP levels [38]. In other words, improving exercise or physical activity can also reduce serum levels of hs-CRP [39, 40]. In addition, participants with a long-distance and a 6-minute walk distance capability (*i.e.*, high functional capacity) had improved survival [41] and reduced levels of inflammatory markers [42]. Therefore, the mechanism linking CRP and functional capacity may involve a reduction in inflammatory markers, thus showing that reduced functional capacity (by a 6-minute walk test) predicted an elevation in CRP levels. This might be in part due to the risk of chronic inflammatory processes linked to the aging process and the development of sarcopenia in older people.

Some limitations should be noted in the present study, as the number of participants was relatively small because of the COVID-19 pandemic in Thailand. However, the prevalence of sarcopenia and its relationship with CRP levels were in agreement with the values reported in several studies of 12.70% and high hs-CRP levels being linked to sarcopenia compared to low hs-CRP levels in older adults. In addition, over 80% of the participants were women with unclear underlying diseases. Apparently, the elevated CRP levels has been shown to be associated with many diseases such as cardiovascular disease, type 2 Diabetes, hypertension and neurodegenerative disorders [15]. Therefore, the results should be interpreted cautiously with regard to sex differences and comorbidities. In future studies, it would be useful to study a larger group of participants with equal sex differences.

## CONCLUSION

The study demonstrated that high hs-CRP levels are associated with slow gait speed and low muscle mass, which in turn lead to the development of sarcopenia in older adults. In addition, decreased functional capacity contributes to the prediction of high hs-CRP levels in older people.

## Figures and Tables

**Table 1:** Characteristics of Thai older adults based upon AWGS 2019 (n=126).

	**Total** **(*N*= 126)**	**Sarcopenia** **(*N*= 16)**	**Non-sarcopenia** **(*N*= 110)**	**95% CI**	***p*-value**
Age (yrs)	68.71±5.49	73.81±7.08	67.97±4.83	3.11 to 8.57	<.001
Sex ^a^	-	-	-	-	.080
Female (*N*=102)	102 (100.00%)	10 (9.80%)	92 (90.20%)	-	
Male (*N*= 24)	24 (100.00%)	6 (25.00%)	18 (75.00%	-	
Skeletal muscle index (kg/m^2^)	5.88±1.17	4.78±0.77	6.07±1.18	-1.90to-0.69	<.001
Gait speed (m/s)	1.21±0.21	1.03±0.24	1.24±0.19	-0.31 to -0.10	<.001
Handgrip strength (kg)	21.72±4.65	20.16±5.46	21.94±4.50	-4.23 to0.67	.153
Walking distance (meters)	341.33±87.74	274.94±95.57	350.98±82.65	-120.70 to -31.40	.001
hs-CRP	2.85±4.09	4.75±5.94	2.57±3.70	0.04 to 4.32	.046

**Note:** ^a^analyzed by using chi-square.

hs-CRP: high-sensitivity C-reactive protein.

**Table 2:** Group difference of the hs-CRP according to functional capacity and AWGS-defined criteria.

	**Hi hs-CRP** **(N= 36)**	**Lo hs-CRP** **(N= 90)**	**95%CI**	**p-value**
Age (yrs)	68.97±5.05	68.611±5.68	-1.7 9 to2.51	.740
sarcopenia ^a^	-	-	1.01 to 8.54	.042
Sarcopenia (N= 16)	8 (50.00%)	8 (50.00%)	-	-
No sarcopenia (N=110)	28 (25.45%)	82 (74.55%)	-	-
Sex ^a^	-	-	0.51 to 3.43	.566
Female (N=102)	28 (27.45%)	74 (72.55%)	-	
Male (N=24)	8 (33.33%)	16 (66.67%)	-	-
Skeletal muscle index (kg/m^2^)	5.63±1.08	6.02±1.26	-0.86to0.08	.102
Gait speed (m/s)	1.11±0.18	1.26±0.21	-0.23 to -0.07	<.001
Handgrip strength (kg)	21.26±4.59	21.90±4.68	0.22 to -0.07	.491
Walking distance (meters)	311.61±87.12	353.22±85.60	-75.19 to-8.03	.016

**Note:** ^a^analyzed by using chi-square.

Hi hs-CRP: high-sensitivity C-reactive protein ≥ 3.0 mg/L; Lo hs-CRP: high-sensitivity C-reactive protein <3.0 mg/L.

**Table 3:** Sex difference of the hs-CRP according to cardiovascular endurance and components of sarcopenia (*i.e.*, skeletal mass index, gait speed and handgrip strength).

**-**	**Female**				**Male**			
**-**	**Hi hs-CRP (*N*=28)**	**Lo hs-CRP (*N*= 74)**	**95%CI**	**p-value**	**Hi hs-CRP** **(*N*= 8)**	**Lo hs-CRP** **(*N*= 16)**	**95%CI**	***p*-value**
Age (yrs)	68.54±4.37	67.91±5.10	-1.53to2.79	.565	70.50±7.09	71.88±7.15	-7.78to5.06	.660
Sarcopenia ^a^	-	-	0.79to11.30	.132	-	-	0.38to17.45	.362
Sarcopenia	5	5	-	-	3	3	-	-
No sarcopenia	23	69	-	-	5	13	-	-
Skeletal muscle index (kg/m^2^)	5.38±0.91	5.88±1.17	-0.98to-0.01	.045	6.49±1.25	6.67±1.47	-1.44to1.08	.768
Gait speed (m/s)	1.10±0.17	1.270.20	-0.25to-0.08	<.001	1.12±0.22	1.20±0.25	-0.30 to0.13	.405
Handgrip strength (kg)	19.48±2.27	20.24±2.74	-1.91to0.40	.199	27.50±5.32	29.60±4.10	-6.15 to1.97	.296
Walking distance (meters)	311.71±89.94	360.72±89.30	-88.39to-9.62	.015	311.25±82.15	318.50±55.70	-65.88 to51.38	.800

**Note:** ^a^analyzed by using chi-square

Hi hs-CRP: high-sensitivity C-reactive protein ≥ 3.0 mg/L; Lo hs-CRP: high-sensitivity C-reactive protein <3.0 mg/L

**Table 4:** Point-biserial correlations between hs-CRP categorized and components of sarcopenia and cardiovascular endurance in older adults.

**-**	**Age** **(*p*-value)**	**SMI** **(*p* -value)**	**HG** **(*p* -value)**	**GS** **(*p* -value)**	**Walking** **(*p* -value)**
hs-CRP categorized	0.070(.433)	-0.133 (.139)	-0.065 (.468)	-0.310 (<.001)	-0.268(.002)

**Note:** hs-CRP: high-sensitivity C-reactive protein; SMI: skeletal mass index; HG: handgrip strength; GS: gait speed.

## LIST OF ABBREVIATIONS

AMPK: AMP-activated Protein Kinase
AWGS: Asian Working Group for Sarcopenia
BIA: Bioelectrical Impedance Analysis
COPD: Chronic Obstructive Pulmonary Disease
CRP: C-Reactive Protein
DEXA: Dual-Energy X-ray Absorptiometry
EWGSOP: European Working Group on Sarcopenia in Older People
hs-CRP: High-sensitivity C-Reactive Protein
LMR: Lymphocyte-to-Monocyte Ratio
NLR: Neutrophil-to-Lymphocyte Ratio
PLR: Platelet-to-Lymphocyte Ratio
rpS6: ribosomal protein S6
SMM: Skeletal Muscle Mass
